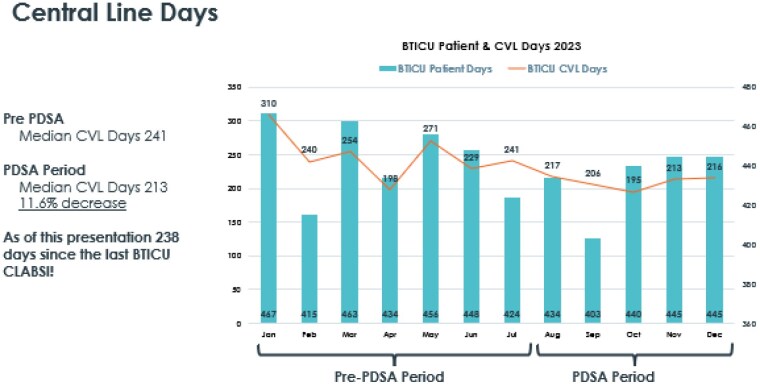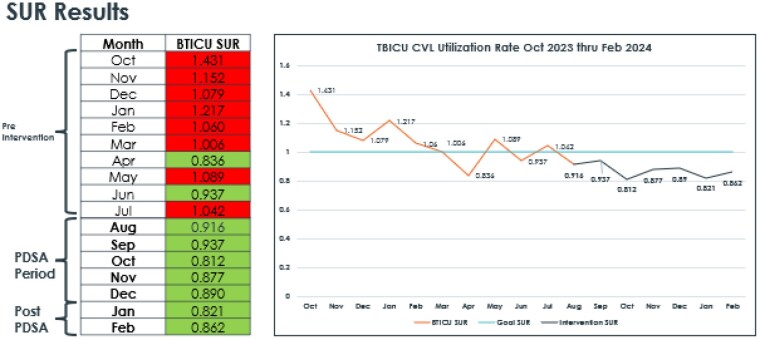# 623 Hardwiring Best Practice to Prevent CLABSIs in the Burn ICU

**DOI:** 10.1093/jbcr/iraf019.252

**Published:** 2025-04-01

**Authors:** Joseph Knapp, Tracy Larson

**Affiliations:** Warden Burn Center; Orlando Health Regional Medical Center

## Abstract

**Introduction:**

Our Burn ICU’s current Standard Utilization Ratio (SUR) of central venous catheters is 1.084on average from 10/22 thru 7/2023 which is above comparable groups. This could lead to increased morbidity, resource utilization and financial strain. Reducing our SUR is crucial to provide higher quality care and improve patient outcomes.

We incurred 4 CLABSIs during FY23, 3 of which were burn patients

Our goal is to reduce our central line utilization ratio from 1.091 to =< 1.0 by the end of the end 2/2024 Furthermore, we wanted to reduce our CVL days by >=10%. To be achieved through evidence-based practice interventions and pragmatic efforts of a multidisciplinary team.

**Methods:**

Throughout this quality improvement process enhanced presence and discussion of CVLs is warranted given the sense of urgency to reduce future CLABSIs from our unique patient population to include all patients admitted to TBICU. Go live date of 8/2023.

* Nurses will review 100% of CVL with MDs during rounds.

* Will monitor peripherally exhausted patients and ensure VAT has assessed patient every 3 days. Including reviews during MDRs

* If providers confirm CVL necessity, ensure indication is accurately documented.

* Staff to notify Charge RN (or CANM/NOM/ANOM) if patient transfers out of TICU with CVL to ensure necessity or alternatives are considered.

* Communicate necessary CVLs, leader to leader when patient transfers out.

* Collaborate with IV experts and HAI team on user of efficacious products for CVL dressings & monitoring

* CLABSI audits by leaders/TBICU RN CLABSI Champions at a reasonable cadence

* Create transfer checklist for patients leaving TBICU with CVL

* Create guidelines for Charge RN to ensure equitable staffing resources for each patient.

**Results:**

Pre Intervention CVL SUR

10/22 - 1.431

11/22 - 1.152

12/22 - 1.079

1/23 - 1.217

2/23 - 1.060

3/23 - 1.006

4/23 - 0.836

5/23 - 1.089

6/23 - 0.937

7/23 - 1.042

PDSA period CVL SUR

8/23 - 0.916

9/23 - 0.937

10/23 - 0.812

11/23 - 0.877

12/23 - 0.890

1/24 - 0.821

Post Intervention CVL SUR

2/24 - 0.862

3/24 - 1.064

4/24 - 0.352

Pre Intervention CVL Days

1/23 - 310

2/23 - 240

3/23 - 254

4/23 - 198

5/23 - 271

6/23 - 229

7/23 - 241

PDSA Period CVL Days

8/23 - 217

9/23 - 206

10/23 - 195

11/23 - 213

12/23 - 216

1/24 - 201

Post Intervention CVL Days

2/24 - 198

3/24 - 216

4/24 - 90

**Conclusions:**

We met our goal of achieving an CVL SUR of <=1.0 with 7 consecutive months of CVL SUR well under 1.00 with an avg 0.875 through the intervention period median CVL days from 241 to 210 days (12.8%) decrease.

Communication, education, vigilance, visible leadership presence and a multifaceted approach to implementation of best practice has led to consistency in achieving best practice and positive patient outcomes.

**Applicability of Research to Practice:**

Best practices are reproduced with positive outcomes through education, active participation in multidisciplinary rounds, effective use of available resources, and listening to concerns of the bedside RN to produce practical solutions to CVL dressing and monitoring.

**Funding for the Study:**

N/A